# Cohort profile: the KDCA-Tuberculosis-NHIS cohort linking tuberculosis surveillance and health insurance data in Korea

**DOI:** 10.4178/epih.e2025071

**Published:** 2025-12-13

**Authors:** Dawoon Jeong, Jinsun Kim, Seung Won Lee, Hongjo Choi, Hojoon Sohn, Jieun Kim, Hyewon Lee, Hyeran Jeong, Seung Eun Lee, Young-joon Park, Jaiyong Kim, Eun Mi Kim, Minji Koo, Hoyeon Jang, Young Ae Kang

**Affiliations:** 1 Department of Preventive Medicine, Seoul National University College of Medicine, Seoul, Korea; 2 Division of Tuberculosis Policy, Department of Infectious Disease Policy, Korea Disease Control and Prevention Agency, Cheongju, Korea; 3 Institute for Immunology and Immunological Disease, Yonsei University College of Medicine, Seoul, Korea; 4 Division of Health Policy and Management, Korea University College of Health Science, Seoul, Korea; 5 Department of Big Data Research and Development, National Health Insurance Service, Wonju, Korea; 6 Division of Pulmonary and Critical Care Medicine, Department of Internal Medicine, Severance Hospital, Yonsei University College of Medicine, Seoul, Korea; 7 Institute for Innovation in Digital Healthcare, Yonsei University, Seoul, Korea

**Keywords:** Tuberculosis, Linkage, K-TB-N cohort

## Abstract

Despite a steady decline in incidence, tuberculosis (TB) remains a substantial public health burden in Korea, particularly among older adults. Existing national TB surveillance systems lack sufficiently comprehensive data to assess long-term outcomes and health disparities. The K-TB-N cohort integrates data from 3 national sources: the Korean Tuberculosis Surveillance System (2011-2022), the National Health Insurance Database (2010-2022), and mortality data from Statistics Korea (2010-2022). After data cleaning and linkage, the final cohort included 373,812 patients (375,440 episodes) with either drug-susceptible TB or drug- resistant TB. TB notifications declined by approximately 60% over the study period, while the median patient age continued to rise. Treatment success improved over time, accompanied by reductions in lost to follow-up. However, mortality during treatment increased, with more than half of deaths attributed to non-TB causes such as pneumonia, cancer, and cardiovascular disease. Post-treatment mortality also remained high, particularly among patients with drug-resistant TB. The K-TB-N cohort provides a comprehensive, linked dataset for advancing research on TB epidemiology, treatment outcomes, comorbidities, and health disparities. It enables evaluations of public health interventions, long-term prognosis, and strategies for post-TB care. This cohort will remain a valuable resource for shaping data-driven TB control policies in aging and high-burden settings.

## INTRODUCTION

Tuberculosis (TB) remains a major global health concern and continues to be one of the leading infectious causes of morbidity and mortality worldwide. According to the World Health Organization (WHO), 10.8 million people were estimated to have developed TB in 2023, and 1.25 million deaths were attributed to the disease [1]. Despite advances in diagnosis, treatment regimens, and prevention strategies, TB persists as a substantial public health burden, particularly in resource-limited settings.

In Korea, TB continues to pose a significant public health challenge, even though the country is a high-income setting with a robust healthcare infrastructure [2]. Korea has the second-highest TB incidence rate among Organization for Economic Cooperation and Development countries [3,4]. National reports indicate that efforts to reduce TB include implementation of the National Strategic Plan for Tuberculosis Control, active case finding, contact tracing, and expanded latent TB infection screening and treatment [5,6]. Although these policies have contributed to a gradual decline in incidence, challenges such as multidrug-resistant TB and a rapidly aging population still impede progress toward elimination.

Integrated databases have become increasingly important in TB research for enhancing understanding of disease epidemiology, evaluating programmatic interventions, and identifying gaps in care delivery [7]. Such databases allow consolidation of information across multiple sources, supporting comprehensive analyses that incorporate demographic, clinical, and socioeconomic factors [8]. The national TB surveillance data have played a key role in monitoring disease trends, identifying high-risk populations, and evaluating public health measures [9,10]. However, because surveillance systems rely on a single data source, they remain limited in their ability to comprehensively analyze factors such as socioeconomic determinants and long-term treatment outcomes.

Recent efforts in Korea to integrate the Korean Tuberculosis Surveillance System (KTB-Surv) into the Korea Tuberculosis Network (KTB-net) and the National Health Insurance Database (NHID) have demonstrated the utility of linked datasets for addressing TB-related challenges. KTB-Surv within KTB-net [10] provides detailed epidemiological information, including diagnostic and treatment outcomes, while NHID [11] offers extensive data on socioeconomic status, healthcare utilization, and comorbidities, supporting comprehensive analyses of health inequities and intervention effects. For example, integration of data from 2011 to 2018 enabled detailed assessments of treatment outcomes, socioeconomic factors, and mortality trends among TB patients [12]. This approach has provided valuable insights into treatment adherence [13], public health intervention effectiveness [14], and TB-related health disparities [15], thereby supplying critical evidence for national TB control strategies.

This cohort profile describes the development and structure of a novel integrated TB database (K-TB-N) that combines 3 national-level datasets in Korea: KTB-Surv within KTB-net, the NHID, and mortality data from Statistics Korea. The primary objectives are to provide a comprehensive resource for investigating TB epidemiology, evaluating the impact of public health interventions, and identifying areas requiring targeted efforts to achieve TB elimination.

## COHORT DESCRIPTION

### Data sources

The K-TB-N cohort combined information from 3 key national datasets in Korea.

#### KTB-Surv [10]

A web-based system, KTB-net, was established in 2000 and is managed by the Korea Disease Control and Prevention Agency. KTB-Surv functions as the national TB registry and has high data completeness, making it the primary source for data linkage. The TB notification form within KTB-Surv includes patient demographic information; diagnostic test results from clinical, radiologic, bacteriologic, and histologic examinations; diagnostic records; treatment information; and data on deaths occurring during treatment, along with treatment outcomes. This study utilized KTB-Surv data from 2011 to 2022.

#### NHID [11]

The NHID is a comprehensive dataset maintained by the National Health Insurance Service (NHIS) and provides information on (1) socio-demographic characteristics (age, gender, household income level, death, and insurance eligibility); (2) types of healthcare service use (inpatient procedures, operations, prescriptions, etc.); (3) diagnostic and classification data based on the International Classification of Diseases (ICD); (4) drug and treatment prescriptions (generic name, quantity, duration, unit price, etc.); and (5) healthcare provider characteristics (location, level, and provider type). This study utilized NHID data from 2010 to 2022.

#### Mortality data from Statistics Korea [16]

These data include cause-of-death records based on the Korean Standard Classification of Diseases (KCD), enabling detailed analyses of mortality trends among patients with TB. This study utilized mortality data from 2010 to 2022.

### Data cleaning and linkage

#### Data cleaning

A comprehensive cleaning process was applied to the K-TB-N cohort to ensure high-quality data for analysis.

##### Identifying TB cases in KTB-Surv

Cases with diagnostic changes in KTB-Surv were removed to maintain accuracy.

##### Handling duplicated TB reports in KTB-Surv

Multiple reports for the same patient were identified and either combined into a single episode or split into separate episodes according to treatment history and the time interval between the end of one report and the start of the next. A 90-day threshold was used to define distinct episodes.

##### Handling missing data

Patients with missing NHID identifying codes, primarily foreigners without eligibility for appropriate health insurance coverage, and those with missing demographic information due to discontinuation of health insurance enrollment, were identified and excluded.

##### Standardization

Diagnosis codes, treatment regimens, and laboratory results were standardized according to ICD-10 codes and national guidelines.

#### Data linkage

**Figure 1.**
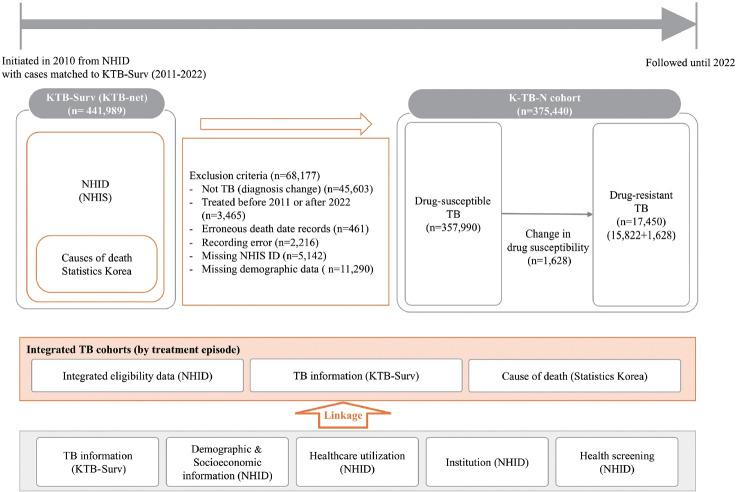
Data linkage of the K-TB-N cohort. KTB-Surv, Korean Tuberculosis Surveillance System; KTB-net, Korea Tuberculosis Network; NHID, National Health Insurance Database; NHIS, National Health Insurance Service; TB, tuberculosis; ID, identification.

Data linkage was performed using anonymized personal identification numbers to ensure privacy (Figure 1). KTB-Surv data from 2011 to 2022 were matched with NHID data from 2010 to 2022 to capture medical histories and socioeconomic information. Mortality data were then integrated to identify death outcomes and provide a comprehensive view of patient trajectories. This multi-source approach enabled detailed tracking of treatment outcomes, long-term prognosis, and factors associated with mortality.

### Cohort characteristics

This cohort comprised patients with TB reported in KTB-Surv between 2011 and 2022, along with linked NHID and mortality data. Inclusion criteria were based on TB diagnosis and treatment information from KTB-Surv, and exclusion criteria included diagnostic changes, non-matching records, and incomplete data. The final cohort consisted of more than 300,000 patients with detailed demographic and clinical profiles. Initially, 441,989 patients with TB were registered between 2011 and 2022. After excluding 68,177 patients (diagnosis change [not TB]: 45,603 cases; treatment before 2011 or after 2022: 3,465 cases; erroneous death dates: 461 cases; recording errors: 2,216 cases; missing NHIS ID numbers: 5,142 cases; and missing demographic data: 11,290 cases), 373,812 TB patients remained in the final cohort. Of these, 357,990 were classified as drug-susceptible TB (DS-TB) and 15,822 as drug-resistant TB (DR-TB). Among the DS-TB cases, 1,628 patients later developed drug resistance and were re-registered as DR-TB during a subsequent episode (Supplementary Material 1).

#### Variables

The K-TB-N cohort included a wide range of variables from 3 national datasets (Table 1, Supplementary Material 2). These variables were categorized into patient characteristics (KTB-Surv and NHID), tuberculosis information (KTB-Surv), healthcare utilization (NHID), health screening information (NHID), and mortality data (NHID and Statistics Korea). Together, these data provide a comprehensive foundation for analyzing TB epidemiology, treatment outcomes, and the effects of socioeconomic factors.

#### Patient and public involvement

To approve the study design, the study protocol was reviewed by an Independent Review Committee operated by the NHIS, which included representatives from civil society. After this approval, there was no public or patient involvement during the implementation of the study.

### Ethics statement

This study protocol was approved by the Institutional Review Board of Severance Hospital (4-2023-0463) and the Korea NHIS Medical Request Review Committee (NHIS-2024-1-187, NHIS-2024-1-188). The requirement for informed consent was waived because this was a retrospective study and all data were anonymized. The study complied with the principles of the Declaration of Helsinki, and all procedures were conducted in accordance with the relevant guidelines.

## KEY FINDINGS

### Descriptive statistics

The demographic and clinical characteristics of the study cohort are presented in Table 2. The number of reported TB cases gradually decreased from 46,139 in 2011 to 17,622 in 2022, reflecting an approximately 60% reduction over the 11-year period. The median age of TB patients increased from 51 years in 2011 to 68 years in 2022, demonstrating a marked aging of the TB population. Among the total cohort (n=375,440), 59.2% were men and 40.8% were women. The proportion of men patients remained consistently higher throughout the study period, ranging from 59.4% in 2011 to 60.4% in 2022. Most TB cases were newly diagnosed (87.6%), while 12.4% were previously treated cases. The proportion of previously treated cases declined from 16.5% in 2011 to 10.2% in 2022.

Regarding drug resistance, most TB cases were drug-susceptible (95.4%), whereas 4.6% were classified as DR-TB. The proportion of rifampin-resistant or multidrug-resistant TB (RR/MDR-TB) cases was 1.9% in 2011 and 2.4% in 2022. Isoniazid-resistant tuberculosis (HR-TB) was identified beginning in 2014, when drug susceptibility testing (DST) data became systematically available; the proportion increased from 2.1% in 2014 to 4.4% in 2022 (Table 2).

### Treatment outcomes and causes of death

#### Treatment outcomes in DS-TB patients

**Figure 2.**
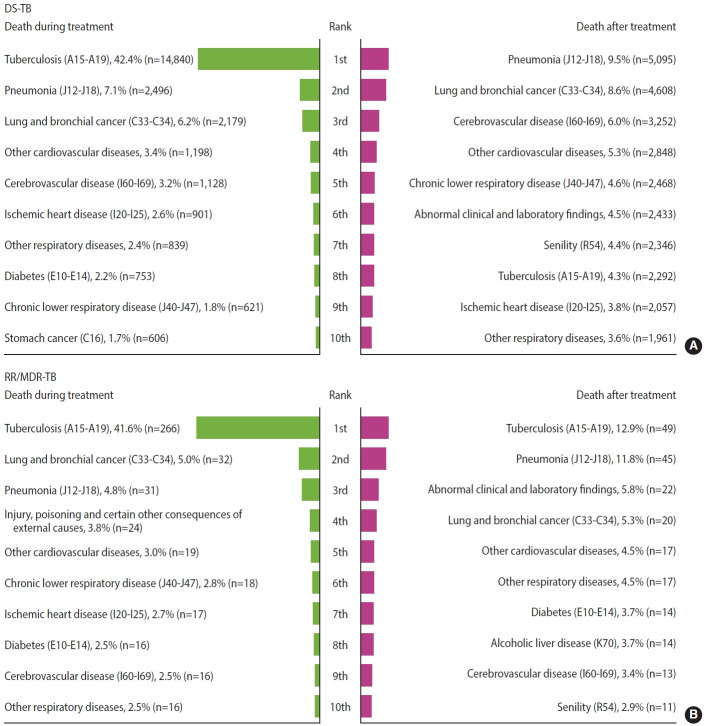
Top 10 causes of death (ICD-10 code) during and after TB treatment among (A) DS-TB and (B) RR/MDR-TB in K-TB-N cohort. DS, drug-susceptible; TB, tuberculosis; RR/MDR, rifampin-resistant/multidrug-resistant; ICD-10, International Classification of Diseases 10th revision.

Treatment outcomes were classified according to WHO definitions and Korean TB treatment guidelines, and are presented in Table 3. Among patients with DS-TB, treatment outcomes from 2011 to 2021 were as follows: treatment success, 83.1%; treatment failure, 0.1%; lost to follow-up (LTFU), 3.3%; not evaluated (NE), 3.7%; and death during treatment, 9.9%. The treatment success rate increased from 76.6% in 2011 to 86.3% in 2015 and then remained relatively stable. The LTFU rate declined from 5.2% in 2011 to 2.4% in 2021, suggesting improved treatment adherence and enhanced program performance. In contrast, mortality during treatment rose sharply from 5.8% in 2011 to 15.3% in 2021. This increase appears to be closely associated with the progressive aging of the TB population in Korea. As the median age increased from 51 years in 2011 to 68 years in 2022, patients presented with a greater burden of comorbidities, likely heightening vulnerability to non–TB-related conditions such as pneumonia, malignancy, and cardiovascular disease. Consequently, more than half of the deaths recorded during TB treatment were attributable to causes other than TB itself (Figure 2). These findings demonstrate the influence of demographic shifts on TB outcomes and highlight the need for integrated approaches that address both TB management and comorbidity care in older adults.

#### Treatment outcomes in HR-TB patients

Treatment outcomes for patients with HR-TB were analyzed from 2014 to 2021 (Table 4). Treatment success was 85.7%, treatment failure 0.1%, LTFU 2.6%, NE 1.2%, and death 10.4%.

#### Treatment outcomes in RR/MDR-TB patients

The treatment outcomes for RR/MDR-TB patients were analyzed from 2011 to 2020 (Table 5). Treatment success was 72.5%, treatment failure 0.9%, LTFU 7.7%, NE 6.6%, and death 12.2%. The treatment success rate varied over time, ranging from 63.0% in 2011 to 73.3% in 2020. The LTFU rate declined markedly from 13.4% in 2011 to 3.0% in 2020, indicating improved patient retention in RR/MDR-TB treatment programs. The mortality rate during treatment increased from 7.2% in 2011 to 18.4% in 2020.

#### Causes of death among TB patients

Mortality data from Statistics Korea were used to classify causes of death among TB patients. The leading causes of death were categorized according to the KCD, which aligns with ICD-10. The top 10 causes of death during and after TB treatment among patients with DS-TB and RR/MDR-TB are shown in Figure 2.

##### Causes of death in DS-TB patients

TB (ICD-10 A15-A19) was the primary cause of death during TB treatment, accounting for 42.4% of deaths. The remaining 57.6% were attributed to non–TB-related causes. Pneumonia (ICD-10 J12-J18) and lung and bronchial cancer (ICD-10 C33-C34) contributed 7.1% and 6.2% of deaths, respectively. Pneumonia was the leading cause of death after treatment completion, representing 9.5% of deaths. Lung and bronchial cancer and cerebrovascular disease (ICD-10 I60-I69) accounted for 8.6% and 6.0%, respectively. Despite successful treatment completion, TB still contributed to 4.3% of deaths after treatment.

##### Causes of death in DR-TB patients

TB (ICD-10 A15-A19) was also the leading cause of death during treatment among patients with DR-TB, accounting for 41.6% of deaths. Lung and bronchial cancer contributed 5.0%, and pneumonia accounted for 4.8%. After treatment completion, TB continued to be a major cause of mortality, responsible for 12.9% of deaths among RR/MDR-TB patients. Pneumonia was the second leading cause at 11.8%. Other major contributors included abnormal clinical and laboratory findings (5.8%), lung and bronchial cancer (5.3%), cardiovascular diseases (4.5%), and other respiratory diseases (4.5%). Even after successful treatment, TB remained a major contributor to mortality in both DS-TB and RR/MDR-TB patients. This likely reflects the chronic sequelae of TB, where post-TB lung disease and systemic comorbidities increase long-term vulnerability. Recent evidence shows that many TB survivors experience persistent symptoms, functional impairment, and progressive complications, all of which may contribute to excess mortality in the post-TB period [17].

## STRENGTHS AND WEAKNESSES

The K-TB-N cohort is the first national-level integrated TB cohort in Korea that combines 3 distinct datasets, addressing the limitations of single-source TB registries. The linkage of clinical data (KTB-Surv), health insurance claims (NHID), and mortality records (Statistics Korea) enables long-term tracking of treatment outcomes and post-treatment mortality. This study provides essential insights into DR-TB-related and TB-related mortality, offering critical evidence to support national TB control strategies and public health policies.

Despite these strengths, the K-TB-N cohort has several limitations. First, missing information in the linkage process may have led to the exclusion of some TB patients. Certain cases recorded in KTB-Surv lacked corresponding NHID medical claims, particularly those treated in public health centers or among foreign nationals. Second, the NHID does not capture non-reimbursed treatments, which may have resulted in incomplete treatment histories, although the proportion of such cases is expected to be minimal. Third, during the early years of the study period, DR-TB cases were identified using ICD-10 codes. Since 2014, however, improvements in DST information within KTB-Surv have enhanced the accuracy and reliability of drug resistance classifications.

## DATA ACCESSIBILITY

The release of data by researchers is not legally permitted. All data are available from the National Health Insurance Sharing Service (NHISS) database (https://nhiss.nhis.or.kr/bd/ay/bdaya001iv.do). NHISS allows data access for any researcher who agrees to comply with research ethics requirements and relevant regulations.

## Figures and Tables

**Table 1.** Composition of K-TB-N cohort variables

Dataset	Component	Variable	Type of variable
KTB-Surv (2011-2022)	TB information	TB history, date of registration, date of treatment commencement, date of treatment outcome, treatment outcome, clinical laboratory test results (radiography, sputum smear test, sputum culture test, PCR, Xpert), PPM medical institution, disease code for TB (ICD-10 code), type of TB, type of medical institution where the patient was registered, use of each TB drug, TB drug resistance	Personal, year
NHID (2010-2022)	Demographic information	Year of birth, gender	Personal, yearly
	Socioeconomic information	Residential area, insurance type, insurance contributions, insurance contribution decile, registered disability status	Personal, yearly
	Healthcare utilization	Diagnosis code, information on medical service, treatment from inpatient, outpatient, and pharmacy visits	Daily
	Institution	Type of institution, location of the provider	Unit of institution
	Health screening	Questionnaire and examination information	Personal, yearly
	Death information	Date of death	Once
Causes of death statistics (2010-2022)	Death information	Cause of death	Once

KTB-Surv, Korea Tuberculosis Surveillance System; NHID, National Health Insurance Database; TB, tuberculosis; PCR, polymerase chain reaction; PPM, public-private mix; ICD-10, International Classification of Diseases 10th revision.

**Table 2.** The demographic and clinical characteristics of the K-TB-N cohort 2011-2022

Characteristics	Total (n=375,440)	2011 (n=46,139)	2012 (n=42,716)	2013 (n=38,448)	2014 (n=37,368)	2015 (n=34,308)	2016 (n=33,097)	2017 (n=30,436)	2018 (n=28,603)	2019 (n=25,699)	2020 (n=21,309)	2021 (n=19,695)	2022 (n=17,622)
Age, median [IQR] (yr)	59 [42-75]	51 [34-69]	53 [36-70]	54 [38-71]	56 [40-73]	57 [41-74]	59 [44-75]	61 [45-76]	63 [48-78]	64 [49-78]	65 [50-79]	66 [52-79]	68 [54-80]
Gender													
Men	222,378 (59.2)	27,389 (59.4)	25,225 (59.1)	22,570 (58.7)	21,868 (58.5)	20,348 (59.3)	19,590 (59.2)	17,888 (58.8)	17,027 (59.5)	15,363 (59.8)	12,720 (59.7)	11,745 (59.6)	10,645 (60.4)
Women	153,062 (40.8)	18,750 (40.6)	17,491 (41.0)	15,878 (41.3)	15,500 (41.5)	13,960 (40.7)	13,507 (40.8)	12,548 (41.2)	11,576 (40.5)	10,336 (40.2)	8,589 (40.3)	7,950 (40.4)	6,977 (39.6)
Newly treated	328,765 (87.6)	38,530 (83.5)	37,101 (86.9)	33,630 (87.5)	33,235 (88.9)	29,968 (87.4)	29,007 (87.6)	26,685 (87.7)	25,133 (87.9)	22,846 (88.9)	19,088 (89.6)	17,712 (89.9)	15,830 (89.8)
Previously treated	46,675 (12.4)	7,609 (16.5)	5,615 (13.1)	4,818 (12.5)	4,133 (11.1)	4,340 (12.7)	4,090 (12.3)	3,751 (12.3)	3,470 (12.1)	2,853 (11.1)	2,221 (10.4)	1,983 (10.1)	1,792 (10.2)
DS-TB	357,990 (95.4)	45,247 (98.1)	42,065 (98.5)	37,648 (97.9)	35,715 (95.6)	32,102 (93.6)	30,952 (93.5)	28,637 (94.1)	26,794 (93.7)	24,015 (93.5)	19,948 (93.6)	18,429 (93.6)	16,438 (93.3)
HR-TB	9,171 (2.4)	2 (0)	19 (0)	85 (0.2)	778 (2.1)	1,343 (3.9)	1,306 (4.0)	1,074 (3.5)	1,107 (3.9)	1,048 (4.1)	867 (4.1)	774 (3.9)	768 (4.4)
RR/MDR-TB	8,279 (2.2)	890 (1.9)	632 (1.5)	715 (1.9)	875 (2.3)	863 (2.5)	839 (2.5)	725 (2.4)	702 (2.5)	636 (2.5)	494 (2.3)	492 (2.5)	416 (2.4)

Values are presented as number (%). TB, tuberculosis; IQR, interquartile range; DS, drug-susceptible; HR, isoniazid-resistant; RR/MDR, rifampin-resistant/multidrug-resistant.

**Table 3.** Treatment outcomes of drug-susceptible tuberculosis, 2011-2021

Outcomes	Total (n=341,552)	2011 (n=45,247)	2012 (n=42,065)	2013 (n=37,648)	2014 (n=35,715)	2015 (n=32,102)	2016 (n=30,952)	2017 (n=28,637)	2018 (n=26,794)	2019 (n=24,015)	2020 (n=19,948)	2021 (n=18,429)
Treatment success	283,755 (83.1)	34,637 (76.6)	34,121 (81.1)	31,501 (83.7)	30,363 (85.0)	27,689 (86.3)	26,609 (86.0)	24,557 (85.8)	22,737 (84.9)	20,204 (84.1)	16,425 (82.3)	14,912 (80.9)
Treatment failure	192 (0.1)	38 (0.1)	38 (0.1)	26 (0.1)	23 (0.1)	13 (0)	9 (0)	7 (0)	7 (0)	10 (0)	8 (0)	13 (0.1)
Lost to follow-up	11,248 (3.3)	2,348 (5.2)	1,889 (4.5)	1,539 (4.1)	1,272 (3.6)	938 (2.9)	745 (2.4)	612 (2.1)	565 (2.1)	483 (2.0)	414 (2.1)	443 (2.4)
Not evaluated	12,669 (3.7)	5,617 (12.4)	3,177 (7.6)	1,813 (4.8)	846 (2.4)	241 (0.8)	156 (0.5)	160 (0.6)	137 (0.5)	129 (0.5)	157 (0.8)	236 (1.3)
Death	33,688 (9.9)	2,607 (5.8)	2,840 (6.8)	2,769 (7.4)	3,211 (9.0)	3,221 (10.0)	3,433 (11.1)	3,301 (11.5)	3,348 (12.5)	3,189 (13.3)	2,944 (14.8)	2,825 (15.3)

Values are presented as number (%).

**Table 4.** Treatment outcomes of isoniazid-resistant tuberculosis, 2014-2021

Outcomes	Total (n=8,297)	2014 (n=778)	2015 (n=1,343)	2016 (n=1,306)	2017 (n=1,074)	2018 (n=1,107)	2019 (n=1,048)	2020 (n=867)	2021 (n=774)
Treatment success	7,108 (85.7)	679 (87.3)	1,172 (87.3)	1,133 (86.8)	924 (86)	942 (85.1)	912 (87)	734 (84.7)	612 (79.1)
Treatment failure	7 (0.1)	1 (0.1)	0 (0)	0 (0)	2 (0.2)	1 (0.1)	2 (0.2)	1 (0.1)	0 (0)
Lost to follow-up	217 (2.6)	31 (4.0)	42 (3.1)	35 (2.7)	33 (3.1)	23 (2.1)	24 (2.3)	16 (1.9)	13 (1.7)
Not evaluated	101 (1.2)	14 (1.8)	17 (1.3)	8 (0.6)	9 (0.8)	9 (0.8)	8 (0.8)	8 (0.9)	28 (3.6)
Death	864 (10.4)	53 (6.8)	112 (8.3)	130 (10.0)	106 (9.9)	132 (11.9)	102 (9.7)	108 (12.5)	121 (15.6)

Values are presented as number (%).

**Table 5.** Treatment outcomes of rifampin-resistant/multidrug-resistant tuberculosis, 2011-2020

Outcomes	Total (n=7,371)	2011 (n=890)	2012 (n=632)	2013 (n=715)	2014 (n=875)	2015 (n=863)	2016 (n=839)	2017 (n=725)	2018 (n=702)	2019 (n=636)	2020 (n=494)
Treatment success	5,347 (72.5)	561 (63.0)	441 (69.8)	532 (74.4)	667 (76.2)	655 (75.9)	611 (72.8)	528 (72.8)	533 (75.9)	457 (71.9)	362 (73.3)
Treatment failure	69 (0.9)	11 (1.2)	11 (1.7)	10 (1.4)	13 (1.5)	7 (0.8)	5 (0.6)	1 (0.1)	6 (0.9)	3 (0.5)	2 (0.4)
Lost to follow-up	569 (7.7)	119 (13.4)	70 (11.1)	61 (8.5)	65 (7.4)	61 (7.1)	59 (7.0)	49 (6.8)	36 (5.1)	34 (5.4)	15 (3.0)
Not evaluated	488 (6.6)	135 (15.2)	52 (8.2)	44 (6.2)	40 (4.6)	34 (3.9)	54 (6.4)	35 (4.8)	36 (5.1)	34 (5.4)	24 (4.9)
Death	898 (12.2)	64 (7.2)	58 (9.2)	68 (9.5)	90 (10.3)	106 (12.3)	110 (13.1)	112 (15.4)	91 (13.0)	108 (17.0)	91 (18.4)

Values are presented as number (%).

## Supplementary materials

Supplementary Material 1. Participants of K-TB-N cohort

Supplementary Material 2. Variable description of K-TB-N cohort
