# Supplementary material for: Cohort profile: the KDCA-Tuberculosis-NHIS cohort linking tuberculosis surveillance and health insurance data in Korea
Source: Epidemiol Health. 2025 Dec 13;47:e2025071. doi: 10.4178/epih.e2025071 (PMC12884042; doi:10.4178/epih.e2025071)
Supplement: Supplementary Material 1. — Participants of K-TB-N cohort [file epih-47-e2025071-Supplementary-1.docx]

Supplementary Material 1. Participants of K-TB-N cohort


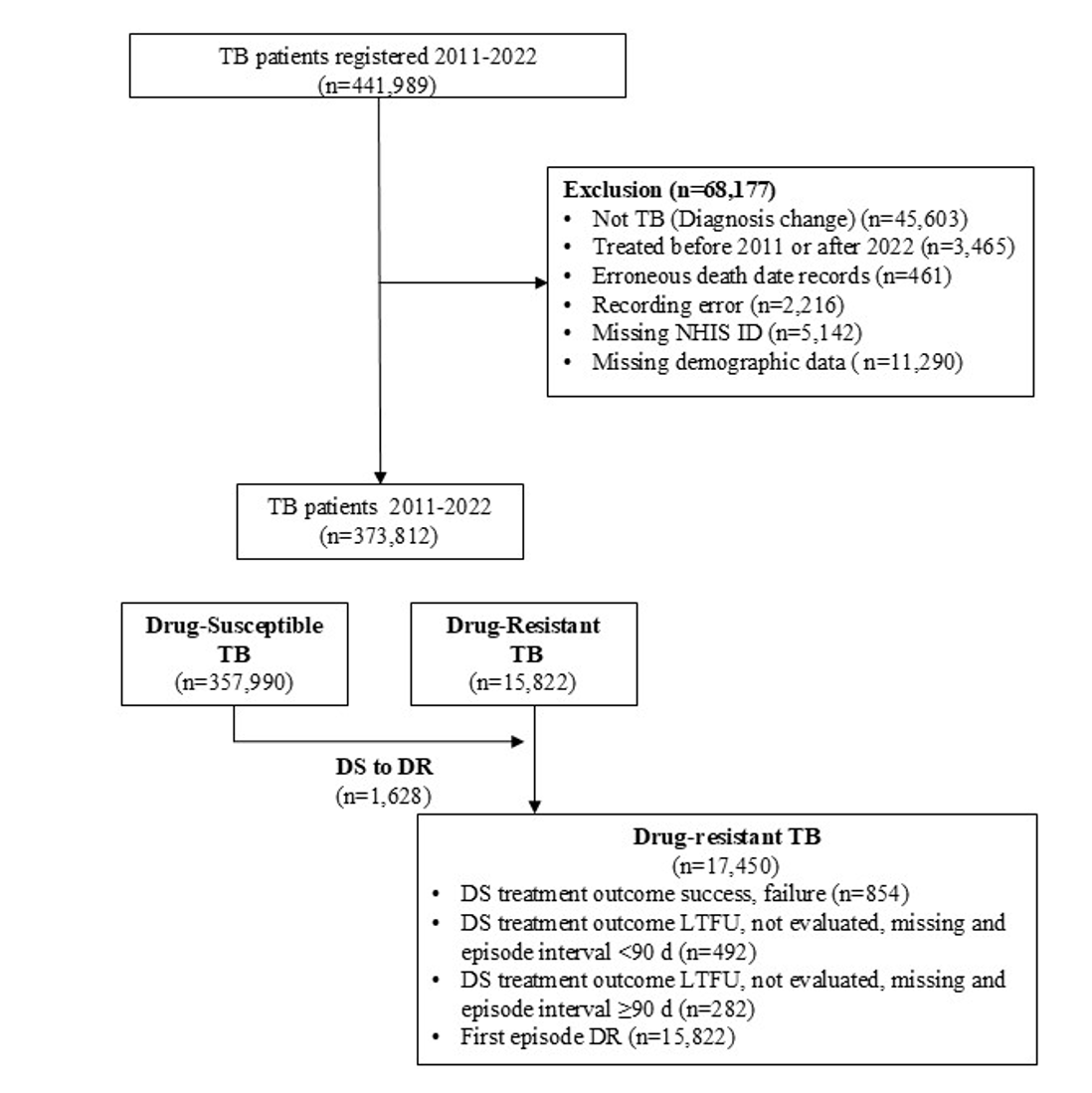


TB; tuberculosis, ID; identification, DS; drug susceptible, DR; drug resistant, LTFU; lost to follow-up,
