# Supplementary material for: Cohort profile: the KDCA-Tuberculosis-NHIS cohort linking tuberculosis surveillance and health insurance data in Korea
Source: Epidemiol Health. 2025 Dec 13;47:e2025071. doi: 10.4178/epih.e2025071 (PMC12884042; doi:10.4178/epih.e2025071)
Supplement: Supplementary Material 2. — Variable description of K-TB-N cohort [file epih-47-e2025071-Supplementary-2.docx]

Supplementary Material 2. Variable description of K-TB-N cohort

| **Dataset** | **Components** | **Variable** | **Description** |
| --- | --- | --- | --- |
| **K-TB-N cohort (2011-2022)** | Integrated eligibility data | Age | Calculated as the difference between the year of tuberculosis treatment initiation and the year of birth. |
|  |  | Gender | Gender recorded in the year of cohort enrollment. |
|  |  | Residential area | Residential area recorded in the year of cohort enrollment. |
|  |  | Insurance type | Type of insurance recorded in the year of cohort enrollment. |
|  |  | Insurance contribution decile | Decile based on the insurance contribution in the year of cohort enrollment. Medicare recipients are categorized as 0; all others are classified according to their insurance contribution decile. |
|  | TB information | TB history | Patients are classified as new cases if recorded as such at least once during the treatment episode. |
|  |  | Date of treatment commencement | The date when tuberculosis treatment was first initiated during the treatment episode. |
|  |  | Date of treatment outcomes | The date when the final treatment outcome was recorded within the treatment episode. |
|  |  | Clinical laboratory test (radiography, sputum test) | Results of laboratory tests conducted within 30 days before or after the treatment initiation date. |
|  |  | PPM medical institution | The medical institution that reported the treatment outcomes during the treatment episode |
|  |  | Type of TB | Classification of tuberculosis as pulmonary or extrapulmonary, based on ICD-10 codes during the treatment episode. |
|  |  | Drug resistance | Isoniazid monoresistance: Resistance to isoniazid while rifampin remains susceptible.  Rifampin monoresistance: Resistance to rifampin while isoniazid remains susceptible.  Multidrug resistance: Resistance to both isoniazid and rifampin. |

TB; tuberculosis, ICD-10 ; International Statistical Classification of Diseases and Related Health Problems 10th Revision
